# Functional Conservation and Genetic Divergence of Chordate Glycinergic Neurotransmission: Insights from Amphioxus Glycine Transporters

**DOI:** 10.3390/cells10123392

**Published:** 2021-12-02

**Authors:** Matteo Bozzo, Simone Costa, Valentina Obino, Tiziana Bachetti, Emanuela Marcenaro, Mario Pestarino, Michael Schubert, Simona Candiani

**Affiliations:** 1 Dipartimento di Scienze della Terra, dell’Ambiente e della Vita (DISTAV), Università degli Studi di Genova, 16132 Genoa, Italy; simone.costa86@gmail.com (S.C.); tiziana.bachetti@unige.it (T.B.); pesta@unige.it (M.P.); 2 Dipartimento di Medicina Sperimentale (DIMES), Università degli Studi di Genova, 16132 Genoa, Italy; valentina.obino@edu.unige.it (V.O.); emanuela.marcenaro@unige.it (E.M.); 3 Laboratoire de Biologie du Développement de Villefranche-sur-Mer (LBDV), Institut de la Mer de Villefranche, Sorbonne Université, CNRS, 06230 Villefranche-sur-Mer, France; michael.schubert@imev-mer.fr

**Keywords:** GlyT, glia, nervous system evolution, central pattern generator, locomotion, cephalochordates

## Abstract

Glycine is an important neurotransmitter in vertebrates, performing both excitatory and inhibitory actions. Synaptic levels of glycine are tightly controlled by the action of two glycine transporters, GlyT1 and GlyT2, located on the surface of glial cells and neurons, respectively. Only limited information is available on glycinergic neurotransmission in invertebrates, and the evolution of glycinergic neurotransmission is poorly understood. Here, by combining phylogenetic and gene expression analyses, we characterized the glycine transporter complement of amphioxus, an important invertebrate model for studying the evolution of chordates. We show that amphioxus possess three glycine transporter genes. Two of these (*GlyT2.1* and *GlyT2.2*) are closely related to *GlyT2* of vertebrates, whereas the third (*GlyT*) is a member of an ancestral clade of deuterostome glycine transporters. *GlyT2.2* expression is predominantly non-neural, whereas *GlyT* and *GlyT2.1* are widely expressed in the amphioxus nervous system and are differentially expressed, respectively, in neurons and glia. Vertebrate glycinergic neurons express *GlyT2* and glia *GlyT1*, suggesting that the evolution of the chordate glycinergic system was accompanied by a paralog-specific inversion of gene expression. Despite this genetic divergence between amphioxus and vertebrates, we found strong evidence for conservation in the role glycinergic neurotransmission plays during larval swimming, the implication being that the neural networks controlling the rhythmic movement of chordate bodies may be homologous.

## 1. Introduction

Glycine is a major inhibitory neurotransmitter in the vertebrate spinal cord, brainstem, and retina, where it activates ionotropic glycine receptors (GlyR) [1,2,3,4]. In addition, glycine acts as a co-agonist of *N*-methyl-D-aspartate (NMDA) glutamate receptors [5,6]. Consequently, glycine has a dual role in the vertebrate central nervous system (CNS), acting as an inhibitory neurotransmitter at glycinergic synapses and as a positive modulator of excitatory glutamatergic synapses. In either case, glycinergic neurotransmission is regulated by clearance of glycine from the synaptic space by high-affinity Na^+^/Cl^−^-dependent glycine transporters belonging to the solute carrier family 6 (SLC6) [7]. SLC6 proteins are widely distributed across eukaryotes, serving as transporters of various signaling molecules in unicellular eukaryotes and fungi and as transporters of monoamine and amino acid neurotransmitters in metazoans [8].

In vertebrates, two genes encode glycine transporters, *GlyT1* and *GlyT2*, corresponding to SLC6 members 9 and 5, respectively [9,10,11,12]. Both glycine transporter genes are alternatively spliced to generate multiple variants. In rodents, the GlyT1 protein has five isoforms: three differing in the N-terminal region (GlyT1a, GlyT1b, and GlyT1c) and two differing at the C-terminal end (GlyT1d and GlyT1e) [13,14,15]. GlyT2 protein isoforms (GlyT2a, GlyT2b, and GlyT2c) differ only in their N-terminal region [9,16,17]. GlyT1 and GlyT2 show different pharmacological and kinetic proprieties [7,18] as well as differential expression in the nervous system. *GlyT1* expression is widespread in the CNS, whereas *GlyT2* is mainly restricted to the hindbrain and spinal cord [19,20]. Differences in glycine transporter subtype expression are also observed at the cell type level: GlyT1 is found on astroglial cells and glutamatergic neurons, whereas GlyT2 localizes to glycinergic presynaptic terminals [5]. The observation that some anti-GlyT1 antibodies predominantly stain glial cells associated with glycinergic synapses in the caudal region of the CNS, whereas others stain both astroglia and glutamatergic neurons throughout the CNS [21], further suggests a differential expression of *GlyT1* isoforms. Taken together, the expression data, combined with knockout experiments [22,23], indicate that GlyT2 is responsible for replenishing the glycine pool of the presynaptic terminal, whereas GlyT1 is responsible for terminating glycine signaling at both glycinergic and glutamatergic synapses.

**Figure 1:**
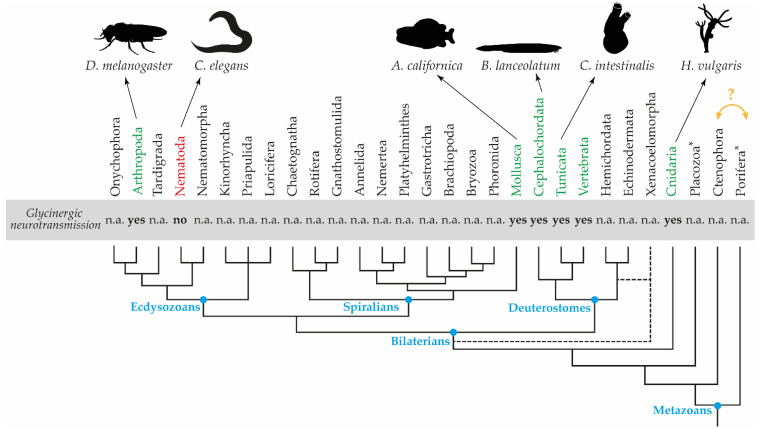
Glycinergic neurotransmission in metazoan animals. Consensus cladogram based on refs [38,39] showing evolutionary relationships of the main animal phyla. The dashed lines indicate that the position of the Xenacoelomorpha is still a matter of debate [40,41,42], which might potentially affect the monophyly of the deuterostomes [43]. The earliest branches of the tree are currently also unresolved, with different phylogenies placing either the Ctenophora or the Porifera as the sister group to all other animals [44,45,46], as indicated by the orange arrow and question mark. Asterisks mark animals without a nervous system. *A. californica*, *Aplysia californica*; *B. lanceolatum*, *Branchiostoma lanceolatum*; *C. elegans*, *Caenorhabditis elegans*; *C. intestinalis*, *Ciona intestinalis*; *D. melanogaster*, *Drosophila melanogaster*; *H. vulgaris*, *Hydra vulgaris*. n.a.: data not available.

Much less is known about glycine transporters, and glycinergic neurotransmission in general, in invertebrates (Figure 1). Indeed, a functional description of glycinergic neurotransmission is only available for the sea hare *Aplysia californica* (phylum Mollusca), a classical neurobiology model, the main invertebrate model organism *Drosophila melanogaster* (phylum Arthropoda), and the tadpole of the sea squirt *Ciona intestinalis* (phylum Tunicata), an important model in evolutionary developmental biology [24,25,26,27]. Conversely, the roundworm *Caenorhabditis elegans* (phylum Nematoda) apparently lacks glycinergic transmission [28]. Importantly, biochemical evidence for the presence of a functional glycine receptor in the freshwater polyp *Hydra vulgaris* (phylum Cnidaria) [29] suggests that glycinergic transmission was already present in the earliest metazoans and subsequently lost in some lineages, such as nematodes. Yet, the tissue-specific distribution of glycine transporters has so far only been investigated in a single invertebrate taxon: *D. melanogaster* [30].

Shpak and coworkers analyzed genomic databases of selected invertebrate deuterostomes (a cephalochordate, an ascidian, and a sea urchin) and retrieved, for each species, two glycine transporter paralogs, which they called *GlyT1-like* and *GlyT2-like*. According to their phylogeny, the *GlyT2-like* genes of invertebrate deuterostomes are *bona fide* orthologs of vertebrate *GlyT2*, whereas the invertebrate *GlyT1-like* genes represent ancestral deuterostome glycine transporters, from which both the *GlyT2* genes of invertebrate deuterostomes and all vertebrate glycine transporter genes evolved [31]. However, the paucity of data on glycinergic neurotransmission among invertebrates makes the evolutionary history of this neurotransmitter difficult to trace. To address this issue, we analyzed the glycine transporters complement of amphioxus, an invertebrate with a key phylogenetic position within the chordates, to define the evolutionary events that took place during the early diversification of vertebrates [32].

Amphioxus (phylum Cephalochordata) is the earliest branch in the chordate lineage, and hence the sister group of the vertebrate plus tunicate clade (Figure 1). Amphioxus has a prototypical chordate body plan with a nervous system consisting of a CNS and a peripheral nervous system (PNS) organized into ectodermal plexuses. The CNS of amphioxus larvae is a hollow tube that is slightly expanded anteriorly to form a cerebral vesicle [33]. Despite lacking overt anatomical landmarks, molecular evidence suggests that the amphioxus CNS is vertebrate-like and regionalized into an anterior hypo-prethalamic, an intermediate dien-mesencephalic, and a posterior rhombospinal region [34]. The neurochemistry of the amphioxus nervous system is similar to that of vertebrates [35,36]. Of particular interest is the presence of evenly-spaced groups of glycinergic neurons in the rhombospinal regions, at the level of somites 2 to 5, with the first two groups also containing GABAergic neurons [35] and with astroglia-like cells being abundant in the whole rhombospinal region [37]. Here, we analyzed the genome of three amphioxus species to characterize the glycine transporter repertoire of cephalochordates. We retrieved additional sequences compared with previous work [31] and report their developmental expression patterns in the amphioxus *Branchiostoma lanceolatum*. Based on an updated phylogeny of deuterostome glycine transporters, this analysis revealed expression of amphioxus glycine transporters in neurons and glial cells, further strengthening the previously proposed homology of amphioxus and vertebrate glial cell populations [37]. However, we found the paralog-specific expression domains of amphioxus and vertebrate glycine transporter genes to be inversed, hinting at a particularly complex evolutionary history of this gene family. Our results nonetheless provide strong support for the evolutionary conservation, at least in chordates, of glycinergic neurotransmission in the control of larval swimming, also highlighting the need for further work on glycine signaling in other invertebrates.

## 2. Materials and Methods

### 2.1. Animals

Sexually mature specimens of the European amphioxus (*Branchiostoma lanceolatum*) were collected at the previously described site in Argelès-sur-Mer, France, transported to the laboratory, and shocked to obtain gametes according to published protocols [47,48]. Embryos were obtained by *in vitro* fertilization, cultured until the desired stage, and fixed with 4% paraformaldehyde in MOPS-EGTA buffer for *in situ* hybridization [47]. Embryos were staged according to Carvalho et al. (2021) [49].

### 2.2. Identification and Cloning of Glycine Transporter Genes from B. lanceolatum

*B. lanceolatum* glycine transporter genes were identified by BLAST searches of transcriptome datasets from this species [50] using the human *GlyT1* and *GlyT2* sequences as queries. The complete sequences of the glycine transporter genes from *B. lanceolatum* were predicted from genomic information extrapolated from EnsemblMetazoa (metazoa.ensembl.org/Branchiostoma_lanceolatum/Info/Index, accessed on 21 October 2021) using the glycine transporter sequences of *B. belcheri* and *B. floridae* [51,52] and the comparative prediction tool FGENESH+ [53]. The positions of the glycine transporter loci in the genomes of three amphioxus species (*B. belcheri*, *B. floridae*, and *B. lanceolatum*) are reported in Table 1.

Total RNA was extracted from *B. lanceolatum* embryos and larvae using the TRIzol Reagent (Thermo Fisher Scientific, Waltham, MA, USA). Reverse transcription was carried out with 1 μg total RNA using the SuperScript III cDNA Synthesis Kit (Thermo Fisher Scientific, Waltham, MA, USA). Partial sequences of *B. lanceolatum* glycine transporter genes were amplified by RT-PCR with specific primers (Supplementary Table S1), cloned into the pCRII-TOPO Vector (Life Technologies, Carlsbad, CA, USA), and used as templates to synthesize sense and antisense riboprobes with the DIG RNA Labeling Kit (Roche, Penzberg, Germany) following the manufacturer’s instructions. To map glycinergic neurons, *VGAT* primers (Supplementary Table S1) were used to clone a partial *VGAT* sequence from *B. lanceolatum*, which was subsequently used to prepare a riboprobe. The *hu-elav* and *glutamic acid decarboxylase* (*GAD*) clones have previously been obtained [54,55].

### 2.3. Phylogenetic Analyses

*B. floridae* and *B. belcheri* glycine transporter proteins were identified by BLAST searches of the NCBI (www.ncbi.nlm.nih.gov, accessed on 21 October 2021), JGI (genome.jgi.doe.gov/Brafl1/Brafl1.home.html, accessed on 21 October 2021), and LanceletDB (genome.bucm.edu.cn/lancelet/, accessed on 21 October 2021) databases. *B. lanceolatum* glycine transporter proteins were predicted as described in Section 2.2. Glycine transporter sequences of other invertebrate and vertebrate species were obtained by database searches using BLASTP and TBLASTX (www.ncbi.nlm.nih.gov/BLAST/, accessed on 21 October 2021). Accession numbers of the sequences used are the following: *Homo sapiens* GlyT1 and GlyT2 (AAB30784 and AAK12641); *Bos taurus* GlyT1 and GlyT2 (DAA30955 and XP_015316662); *Gallus gallus* GlyT1 and GlyT2 (NP_001026450 and XP_015141730); *Xenopus laevis* GlyT1 and GlyT2 (NP_001104228 and NP_001154864); *Danio rerio* GlyT1 and GlyT2 (AAY55909 and NP_001009557); *Petromyzon marinus* GlyT1 and GlyT2 (XP_032822036.1 and XP_032833846); *Ciona intestinalis* GlyT and GlyT2 (XP_002126915 and XP_002127521); *Strongylocentrotus purpuratus* GlyT and GlyT2.1/GlyT2.2 (XP_030836434.1 and XP_783036/XP_780120); *Acropora millepora* GlyT-likea and GlyT-likeb (XP_029190597 and XP_029190596), *Caenorhabditis elegans* neurotransmitter transporter snf11 (NP_505873.2).

**Figure 2:**
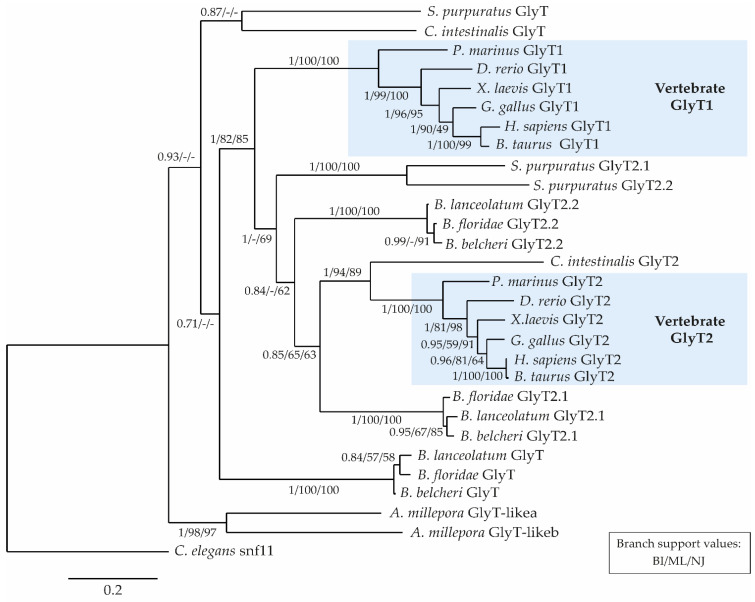
Phylogenetic tree of glycine transporter proteins. Bayesian Inference (BI), Maximum Likelihood (ML), and Neighbor Joining (NJ) methods were used, and the BI tree is shown with branch support values for the BI, ML, and NJ analyses, respectively. “-“ indicates branches not recovered by the corresponding tree reconstruction method. Branch lengths are expressed as amino acid substitutions per site. *Caenorhabditis elegans* neurotransmitter transporter snf11 was used as the outgroup. List of animal species featured in the tree: *Acropora millepora* (coral, cnidarian), *Bos taurus* (cattle, mammal), *Branchiostoma belcheri* (Chinese amphioxus, cephalochordate), *Branchiostoma floridae* (Florida amphioxus, cephalochordate), *Branchiostoma lanceolatum* (European amphioxus, cephalochordate), *Caenorhabditis elegans* (roundworm, nematode), *Ciona intestinalis* (sea squirt, tunicate), *Danio rerio* (zebrafish, teleost fish), *Gallus gallus* (chicken, bird), *Homo sapiens* (human, mammal), *Petromyzon marinus* (sea lamprey, jawless vertebrate), *Strongylocentrotus purpuratus* (purple sea urchin, echinoderm), *Xenopus laevis* (African clawed frog, amphibian).

Protein sequences were aligned using the MUSCLE algorithm in MEGA6 [56]. The alignment used to generate Figure 2 is provided in FASTA format as Supplementary File S1. The multiple sequence alignment was uploaded to the Phylogeny.fr platform [57], and positions with gaps were removed. A first phylogenetic tree was calculated using the Bayesian Inference (BI) method as implemented in the MrBayes program (v3.2.6) [58]. The number of substitution types was fixed to 6. A Poisson model was used for amino acid substitution, whereas rate variation across sites was fixed to “invgamma”. Four Markov Chain Monte Carlo (MCMC) chains were run for 10,000 generations, sampling every 10 generations, with the first 250 sampled trees discarded as “burn-in”. A 50% majority rule consensus tree was constructed. Support for internal branches was evaluated by calculating posterior probabilities. The tree was graphically rendered with TreeDyn (v198.3) [59]. The evolutionary history of glycine transporter sequences was further inferred using the maximum likelihood (ML) and neighbor-joining (NJ) methods in MEGAX [60]. The most appropriate evolutionary models for phylogenetic inference were identified using the Model Selection tool implemented in MEGAX, selecting models with the lowest BIC scores. For the ML tree, a JTT model with Gamma distribution (JTT + G) was the best fit, and the rate variation among sites was modeled with a gamma distribution using a shape parameter of 0.75 in an analysis of 33 amino acid sequences. All positions containing gaps and missing data were eliminated. A total of 538 positions were used in the final dataset. For the NJ tree, evolutionary distances were computed using the same JTT matrix-based method. For both the ML and NJ analyses, support of internal branches was evaluated by 1000 replicates of non-parametric bootstrapping.

### 2.4. Whole Mount In Situ Hybridization

The expression patterns of amphioxus glycine transporter genes were determined by whole mount *in situ* hybridization according to previously published methods [47]. Stained whole mount embryos were mounted with glycerol and photographed using an IX71 inverted microscope (Olympus, Hamburg, Germany) equipped with a ColorViewII camera (Olympus, Hamburg, Germany). Subsequently, selected embryos were retrieved from the slides, rinsed with distilled water, counterstained with 1% Ponceau S in 1% acetic acid, dehydrated with an ethanol series, and embedded in Spurr’s resin (Sigma-Aldrich, St. Louis, MO, USA) [61]. Serial 3 µm sections were obtained using an RM2145 microtome (Leica, Wetzlar, Germany) equipped with a glass knife, mounted in mineral oil, and photographed. For double-label *in situ* hybridization, embryos were hybridized simultaneously with two probes labeled with digoxigenin or fluorescein following published protocols [61]. Staining was performed, respectively, with NBT/BCIP (Roche, Penzberg, Germany,) or Fast Red (Sigma-Aldrich, St. Louis, MO, USA) according to the manufacturers’ instructions. Whole mount embryos were mounted with glycerol and photographed using an IX71 inverted microscope (Olympus, Hamburg, Germany) equipped with a ColorViewII camera (Olympus, Hamburg, Germany) both in brightfield and in epifluorescence using a Texas Red filter to visualize fluorescent Fast Red staining. Selected embryos were retrieved from the slides, rinsed with distilled water, incubated in 20% sucrose overnight, embedded in Killik (Bio-Optica, Milan, Italy), and frozen in liquid nitrogen. Cross-sections were obtained using a CM1900UV cryostat (Leica, Wetzlar, Germany), thawed to room temperature, rinsed in PBS, mounted in 20% glycerol, and photographed.

## 3. Results

### 3.1. Identification and Phylogenetic Analysis of Amphioxus Glycine Transporter Genes

We identified three orthologs of vertebrate glycine transporters in cephalochordates (Table 1), two of which have previously been identified in *B. floridae* and named *GlyT1-like* and *GlyT2-like* [31]. However, based on our phylogenetic reconstructions (Figure 2), the name of the *GlyT1-like* gene was changed to *GlyT*. The phylogenetic tree further demonstrated that the GlyT, GlyT2.1 and GlyT2.2 sequences from *B. lanceolatum, B. floridae*, and *B. belcheri* are homologous (Figure 2), a notion supported by an alignment of the amino acid sequences of the different glycine transporters from these three amphioxus species (Supplementary Table S2). We further found that, at least in *B. floridae* and *B. lanceolatum*, the *GlyT2.1* and *GlyT2.2* genes were located on the same scaffold in the genome and only about 100 kb apart (Table 1), suggesting that the amphioxus *GlyT2.1* and *GlyT2.2* genes originated from a lineage-specific duplication event. Interestingly, two *GlyT2* paralogs were also identified in sea urchins, but not ascidian tunicates or vertebrates (Figure 2).

In the tree, the vertebrate GlyT1 and GlyT2 sequences were clearly separated, falling into two distinct and very strongly supported clades (Figure 2). The amphioxus GlyT2.1 and GlyT2.2 sequences were basal to the ascidian tunicate plus vertebrate GlyT2 sequences, and the echinoderm GlyT2.1 and GlyT2.2 sequences were basal to all chordate GlyT2 sequences. In accordance with previously published phylogenies [31], our phylogenetic analysis revealed that invertebrate deuterostomes lack true GlyT1 orthologs, but rather possess GlyT-like sequences that branch at the base of the clade composed of the GlyT2s and the vertebrate-only GlyT1s. We propose to refer to all members of this group as GlyTs (Figure 2).

### 3.2. Expression of GlyT, GlyT2.1, and GlyT2.2 in Developing Amphioxus

**Figure 3:**
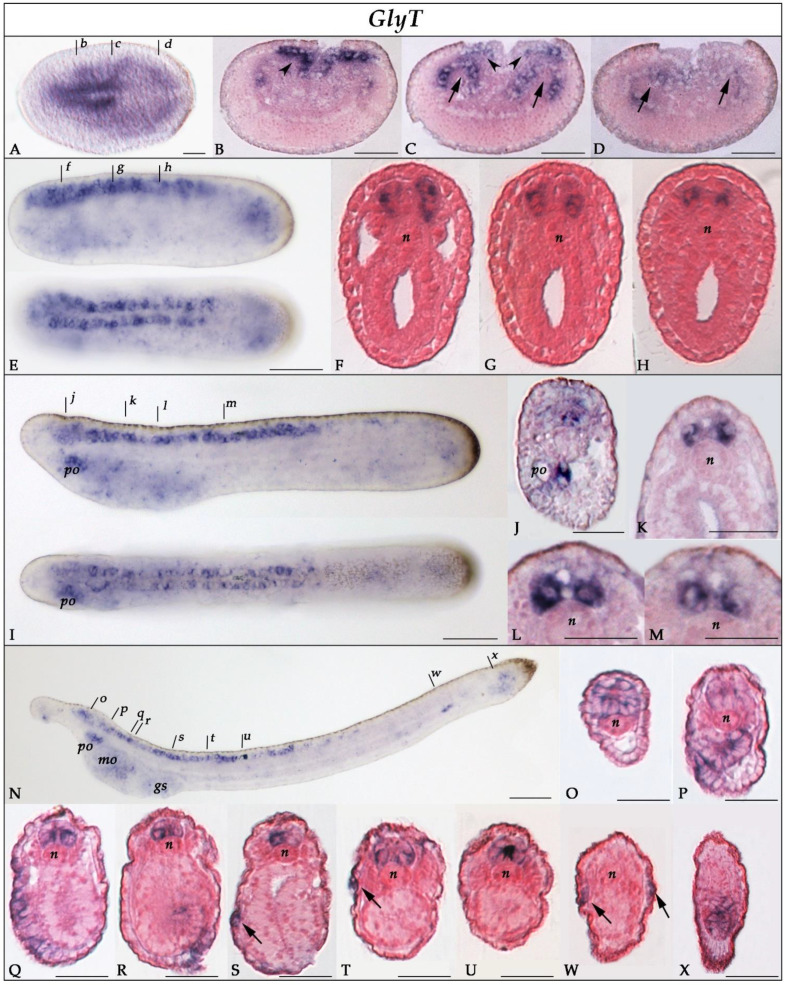
Expression of *GlyT* in developing amphioxus. (**A**) Whole mount N1-stage neurula in dorsal view, anterior to the left. (**B**–**D**) Cross-sections of a N1 neurula at levels indicated in (**A**) by the corresponding lowercase letters. *GlyT* is expressed in the anterior neural plate (arrowheads in **B**,**C**) and in the forming somites (arrows in **C**,**D**). (**E**) Whole mount N4-stage neurula in lateral (top) and dorsal (bottom) view, anterior to the left. (**F**–**H**) Cross-sections of a N4 neurula at levels indicated in (**E**) by the corresponding lowercase letters. *GlyT*-expressing cells are arranged in two lateral columns in the nerve cord. (**I**) Whole mount T1-stage embryo in lateral (top) and dorsal (bottom) view, anterior to the left. (**J**–**M**) Cross-sections of a T1 embryo at levels indicated in (**I**) by the corresponding lowercase letters. *GlyT* is mainly expressed in the cerebral vesicle (**J**) and ventrolateral cells of the anterior two-thirds of the rhombospinal region of the nerve cord (**K**–**M**). At this stage, transcription commences in the developing preoral organ (**I**,**J**). (**N**) Whole mount L1-stage larva in lateral view, anterior to the left and dorsal side up. (**O**–**X**) Cross-sections of a L1 larva at levels indicated in (**N**) by the corresponding lowercase letters. *GlyT* is expressed in ventrolateral cells of the rhombospinal region of the nerve cord (**Q**–**U**) as well as in the preoral organ (**P**). In addition, expression is detectable in the most rostral region of the cerebral vesicle, at the level of the developing frontal eye complex (**O**), in the oral region (**Q**), in the gill slits (**R**), and in ectodermal sensory neurons (arrows in **S**,**T**,**W**). *GlyT* transcripts are also present in the caudal region of the larva at the level of the neurenteric canal (**X**). Abbreviations: gs: gill slit; mo: mouth; n: notochord; po: preoral organ. Scale bars are 50 µm for whole mounts and 25 µm for sections.

In early neurulae (N1 stage), *GlyT* was broadly expressed in the anterior two-thirds of the neural plate and the forming somites (Figure 3A–D). In N4 neurulae, expression was mostly restricted to the CNS (Figure 3E) and particularly to lateral cells of the cerebral vesicle and anterior rhombospinal region (Figure 3F–H). A faint signal was also observed in the pharyngeal and caudal regions. In T1-stage embryos, *GlyT* was expressed in the cerebral vesicle (Figure 3I–J) and in ventrolateral cells of the rhombospinal region up to the end of somite 6 (Figure 3I,K–M). *GlyT* started also to be expressed in the developing preoral organ. In L1 larvae (Figure 3N–X), *GlyT* transcripts were present in the most anterior part of the cerebral vesicle at the level of the frontal eye complex (Figure 3N,O), which at this stage contains glutamatergic and glycinergic neurons [62,63], and in pairs of ventrolateral cells of the rhombospinal region of the nerve cord (Figure 3Q–U). Outside the CNS, *GlyT* was expressed in the preoral organ (Figure 3N,P), in the ectoderm of the oral region (Figure 3Q,R), in the primordium of the first gill slit (Figure 3N,S), and in some ectodermal sensory cells of the trunk (Figure 3S,T,W). Finally, expression was also detected around the neurenteric canal (Figure 3N,X).

**Figure 4:**
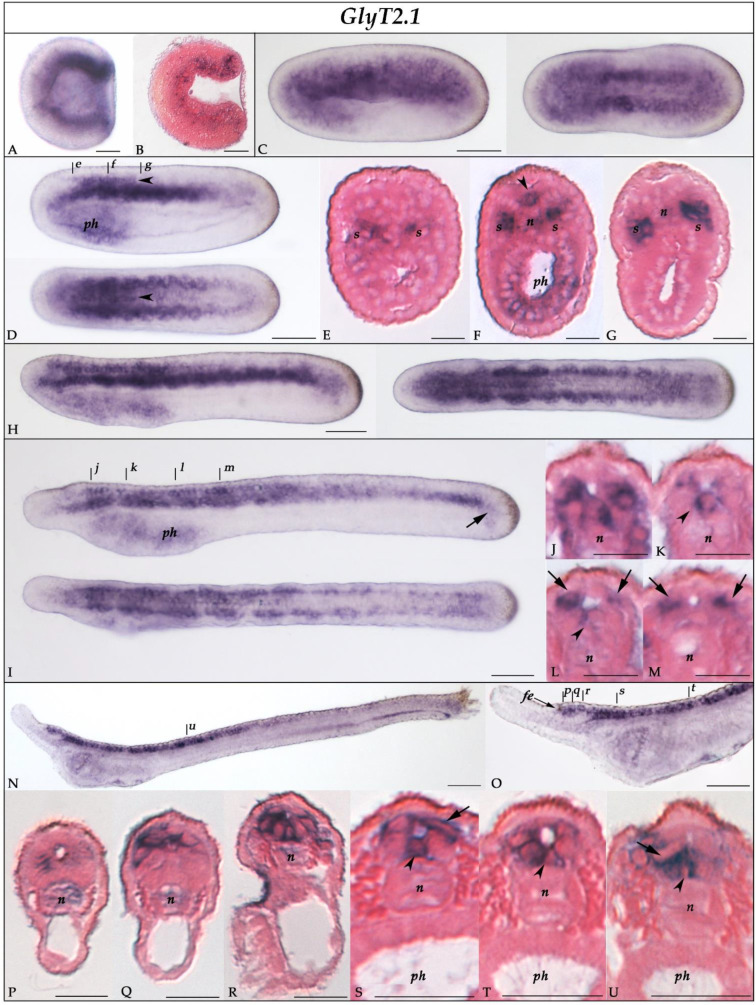
Expression of *GlyT2.1* in developing amphioxus. (**A,B**) G5-stage gastrula in lateral view (**A**), anterior to the left and dorsal side up, and in parasagittal section (**B**). (**C**) Whole mount N1-stage neurula in lateral (left) and dorsal (right) view, anterior to the left. (**D**) Whole mount N4-stage neurula in lateral (top) and dorsal (bottom) view, anterior to the left. (**E**–**G**) Cross-sections of a N4 neurula at levels indicated in (**D**) by the corresponding lowercase letters. *GlyT2.1* is expressed in the somites, pharyngeal endoderm, and floor plate of the anterior rhombospinal region of the nerve cord (arrowheads in **D**,**F**). (**H**) Whole mount T0-stage embryo in lateral (left) and dorsal (right) view, anterior to the left. (**I**) Whole mount T1-stage embryo in lateral (top) and dorsal (bottom) view, anterior to the left. (**J**–**M**) Cross-sections of the nerve cord of a T1 embryo at levels indicated in (**I**) by the corresponding lowercase letters, showing *GlyT2.1* expression in floor plate (arrowheads) and mediolateral ependymal cells (arrows) of the rhombospinal region. Outside the CNS, *GlyT2.1* transcripts are found in the somites, pharyngeal region, and tail bud (**I**, arrow). (**N**) Whole mount L1-stage larva in lateral view, anterior to the left and dorsal side up. (**O**) Enlargement of the anterior part of the embryo presented in N. (**P**–**U**) Cross-sections of a L1 larva at levels indicated in (**N**) and (**O**) by the corresponding lowercase letters. *GlyT2.1* is expressed in the anterior part of the notochord (**P**–**R**), in the cerebral vesicle (**P**–**R**), and in floor plate (arrowheads), ependymal (arrows), and ventrolateral cells of the rhombospinal nerve cord (**S**–**U**). Abbreviations: n: notochord; ph: pharynx; s: somites. Scale bars are 50 µm for whole mounts and 25 µm for sections.

*GlyT2.1* transcripts were first detected in the G5 gastrula around the blastopore and in the presumptive notochord and endoderm (Figure 4A,B). In neurulae (stages N1–N4), *GlyT2.1* was expressed in the anterior rhombospinal region of the neural plate at the level of the floor plate (Figure 4D,F), in the somites (Figure 4C–G), anterior notochord, and pharyngeal endoderm (Figure 4C,F). At the T0/T1 stage, *GlyT2.1* expression was similar to what was observed at previous stages, with expression domains in the anterior nerve cord, somites, and pharyngeal endoderm (Figure 4H–J). Cross-sections of the rhombospinal region of the nerve cord (Figure 4K–M) showed that *GlyT2.1* was expressed by floor plate and ventrolateral ependymal cells [37]. In L1 larvae, *GlyT2.1* was faintly expressed in dorsal cells located posteriorly to the frontal eye complex (Figure 4N–Q) and more strongly in the post-infundibular region of the cerebral vesicle (Figure 4O,R). The expression domain extended along the anterior two-thirds of the rhombospinal nerve cord, predominantly in floor plate (Figure 4S–U) and mediolateral cells (Figure 4S,U). Outside of the CNS, *GlyT2.1* expression in L1 larvae was restricted to the most anterior part of the notochord (Figure 4O–R) and was thus largely downregulated in the pharyngeal region (Figure 4N).

**Figure 5:**
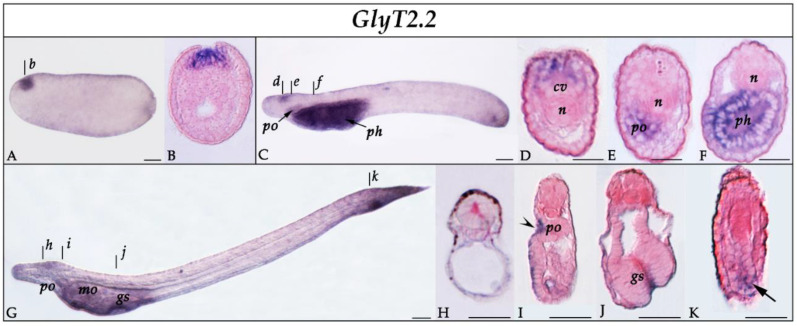
Expression of *GlyT2.2* in developing amphioxus. (**A,B**) N1-stage neurula in whole mount (**A**) and cross-section (**B**) at level indicated in (**A**) by the corresponding lowercase letter. *GlyT2.2* is expressed in the anterior neural plate, corresponding to the prospective cerebral vesicle. (**C**) Whole mount T1-stage embryo in lateral view, anterior to the left. (**D**–**F**) Cross-sections of a T1 embryo at levels indicated in (**C**) by the corresponding lowercase letters. *GlyT2.2* transcripts are detectable in the dorsal part of the cerebral vesicle (**D**), the preoral organ (**E**), and the pharyngeal endoderm (**F**). (**G**) Whole mount L1-stage larva. (**H**–**K**) Cross-sections of an L1 larva at levels indicated in (**G**) by the corresponding lowercase letters. *GlyT2.2* is faintly expressed in the cerebral vesicle (**H**), the preoral organ (**I**, arrowhead), the gill slit primordium (**J**), and the neurenteric canal (**K**, arrow). Abbreviations: cv: cerebral vesicle; gs: gill slit; mo: mouth; n: notochord; ph: pharynx; po: preoral organ. All whole mounts are in lateral view with anterior to the left and dorsal side up. Scale bars are 50 µm for whole mounts and 25 µm for sections.

*GlyT2.2* expression was first detectable at the neurula stage (N1), where it was restricted to a small region of the anterior neural plate (Figure 5A-B), corresponding to the prospective cerebral vesicle. In T1-stage embryos, *GlyT2.2* expression was maintained in the anterior cerebral vesicle (Figure 5C–D) and extended to the preoral organ (Figure 5C,E) and pharyngeal endoderm (Figure 5C,F). In L1-stage larvae, expression in the anterior part of the animal was largely consistent with that observed at the T1 stage, and a new domain of *GlyT2.2* expression appeared around the neurenteric canal (Figure 5G–K).

### 3.3. Glycine Transporter Expression and the Neurochemistry of the Developing Amphioxus Nervous System

**Figure 6:**
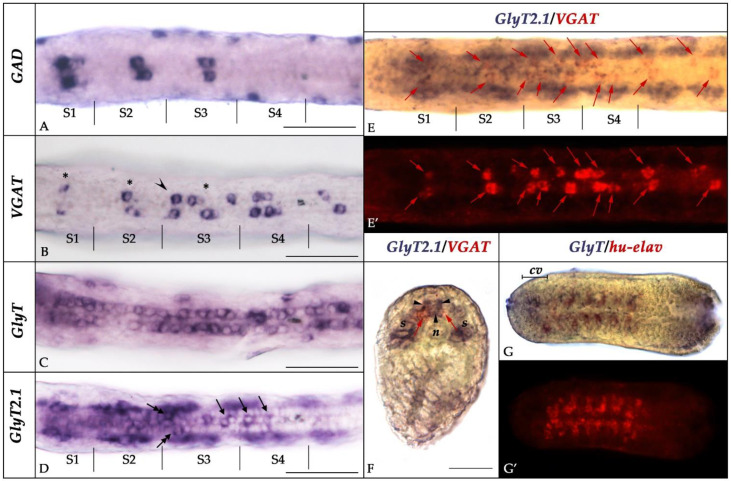
Position of cells expressing glycine transporter genes and neurochemical markers. (**A**–**D**) Dorsal view of the posterior cerebral vesicle and anterior rhombospinal region of T1-stage embryos showing expression of *GAD*, *VGAT*, *GlyT*, and *GlyT2.1* with indicated position of the boundaries of the anteriormost somites (S1, S2, S3, S4). Asterisks in (**B**) indicate *GAD*-expressing cells, whereas arrowheads indicate *GAD*-negative/*VGAT*-positive cells. In (**D**), arrows indicate *GlyT2.1*-positive cells along the midline, whereas double-headed arrows highlight cells that are located more laterally than those expressing *GAD*, *VGAT* or *GlyT*. (**E**–**E’**) T1-stage embryo stained for *GlyT2.1* (purple) and *VGAT* (red) viewed in brightfield (**E**) and fluorescence (**E’**). Enlargement of the same region as shown in (**A**–**D**). Red arrows indicate cells expressing *VGAT*. (**F**) Cross-section of the embryos shown in (**E**), at the level of somite 2. *VGAT* (red arrows) and *GlyT2.1* (arrowheads) are not co-expressed in the same cells. (**G**–**G’**) N4-stage neurula stained for *GlyT* and *hu-elav* viewed in brightfield (**G**) and fluorescence (**G’**). *GlyT* and *hu-elav* are co-expressed in the rhombospinal region, but not in the anterior cerebral vesicle. Abbreviations: cv: cerebral vesicle; n: notochord; s: somite. The scale bar is 25 µm in (**F**) and 50 µm in all other panels.

**Figure 7:**
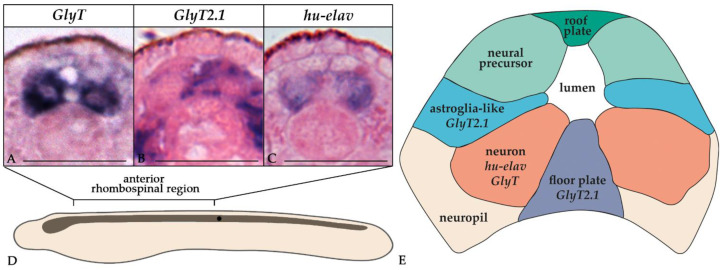
Differential expression of *GlyT* and *GlyT2.1* in neurons and glia in developing amphioxus. (**A**–**C**) Cross-sections of the anterior rhombospinal region of the nerve cord of a T1-stage embryo stained for *GlyT, GlyT2.1*, and *hu-elav*. (**D**) Diagram of a T1-stage embryo with the nerve cord highlighted. The black dot marks the position of the first pigment spot in the nerve cord. (**E**) Diagrammatic representation of a typical cross-section of the rhombospinal region of the nerve cord of a T1-stage amphioxus embryo (adapted from [37]). Neurons, identified by the expression of *hu-elav*, are mostly limited to ventrolateral positions, whereas mediolateral cells and ventral cells are glial cells. Scale bars are 25 µm.

To assign an identity to the cells expressing *GlyT*, *GlyT2.1*, and *GlyT2.2* in the CNS of amphioxus embryos, we compared the expression of the three genes to that of known markers of neural subpopulations, namely *glutamic acid decarboxylase* (*GAD*), the *vesicular GABA/glycine transporter* (*VGAT*), and the pan-neuronal marker *hu-elav* [35,64]. At the T1 stage, the nerve cord contained three pairs of GABAergic neurons (defined by the co-expression of *GAD* and *VGAT* [35]), with each pair being in register with one of the three anteriormost somite pairs (Figure 6A). In the proximity of these pairs of GABAergic neurons, we found cells expressing *VGAT*, but not *GAD* (Figure 6B). These cells have previously been interpreted as glycinergic neurons [35]. Of the three amphioxus glycine transporter genes, *GlyT* (Figure 3) and *GlyT2.1* (Figure 4) were broadly expressed in the nerve cord, whereas *GlyT2.2* expression was limited to the anterior cerebral vesicle (Figure 5). Cells expressing *GlyT* formed two rows running along the anterior-posterior axis of the CNS located adjacent to the floor plate (Figure 6C) in a ventrolateral position (Figure 3K–M). *GlyT* expression was co-localized with that of *hu-elav* (Figure 6G,G’), indicating that *GlyT* is expressed in neurons. Conversely, we found *GlyT2.1* to be expressed in the floor plate as well as in cells lateral to those expressing *GlyT* (Figure 4K–M and Figure 6D). These *GlyT2.1*-expressing cells are glial cells (Figure 7A–E) with marked similarities to vertebrate astroglia [37]. Furthermore, some cells expressing *GlyT2.1* were in the proximity of cells expressing *VGAT* (Figure 6E,E’,F). However, the two signals did not overlap (Figure 6E,E’,F), suggesting that cells of the developing amphioxus CNS do not co-express *GlyT2.1* and *VGAT*.

## 4. Discussion

### 4.1. Evolution of Glycine Neurotransmission in Metazoan Animals

Glycine is an important inhibitory neurotransmitter in the nervous system of vertebrates, where it has been investigated in various species [35,65,66,67,68,69]. Conversely, our understanding of glycinergic neurotransmission in invertebrates is much more limited (Figure 1). Among invertebrates, evidence for glycinergic neurotransmission was first documented in the gastropod mollusk *A. californica*, and data from this model still provide the only information on glycinergic signaling in spiralian animals, showing that glycine is required for the contraction of the ventral aorta. Subsets of neurons innervating the aorta of *A. californica* possess a sodium-dependent glycine uptake system, an axonal glycine transport system, and glycine-containing vesicles in their nerve terminals [24,25]. Interestingly, glycinergic neurotransmission is thought also to modulate blood pressure in vertebrates, but with the inverse role of decreasing contractions and, likely, a different mechanism of action [70].

More recently, glycine was identified as an inhibitory neurotransmitter in an ecdysozoan animal, the fruit fly *D. melanogaster*, where it regulates the duration of a complete circadian cycle [26]. Intriguingly, several lines of evidence strongly suggest that glycine is also involved in the regulation of biological cycles in vertebrates. In zebrafish (*Danio rerio*), glycine and GlyT2 are present in a subset of photoreceptor cells of the pineal organ (called pinealocytes) [71], a neuroendocrine structure that regulates circadian and seasonal behaviors. In zebrafish, the pineal organ projects to mesencephalic and diencephalic structures [72], and some of the projecting fibers are glycinergic [73]. GlyT2 and VGAT are also co-expressed in rat pinealocytes, which release glycine in response to depolarization *in vitro* [74,75]. However, glycine-immunoreactive cells were not reported in the pineal organs of either lampreys, catsharks or sturgeons [65,69,76,77]. Glycine thus appears to be used as a neurotransmitter and, possibly, a paracrine signal in the pineal organ of at least some vertebrate species [71]. Amphioxus larvae perform diurnal migration in the water column, a typical circadian behavior, and possess a putative pineal homolog in the dorsal part of the dien-mesencephalic region: the lamellar body [78,79,80]. The lamellar body consists of two parallel rows of lamellate cells whose cilia form elaborated stacks of membranous lamellae [78]. The number of lamellate cells increases during larval development, but then the organ disaggregates, with only scattered lamellate cells being found in the adult [81]. In N4 neurulae, *GlyT2.1* was faintly expressed in the dorsal part of the prospective posterior cerebral vesicle, where the lamellar body will develop. Interestingly, this stage corresponds to the initiation of lamellar body differentiation [80,82]. During subsequent development (at T0 and T1 stages), *GlyT2.1* expression remained conspicuous in this region of the cerebral vesicle and, in L1 larvae, in which the first lamellate cells have already differentiated [83], *GlyT2.1* expression was still detectable in the lamellar body, extending also to the dorsal part of the anterior cerebral vesicle. These observations hint at a conserved ancestral function of glycine neurotransmission in the pineal organ of chordates.

Genomic analyses have suggested that glycine does not function as a neurotransmitter in the roundworm *C. elegans*, a nematode and thus another ecdysozoan. The *C. elegans* genome contains a vesicular transporter for GABA and glycine (*VGAT/unc-47*), but no obvious orthologs of glycine receptors or transporters, although it does contain a GABA transporter ortholog (*snf-11*) [84]. However, *VGAT/unc-47* is expressed not only in GABAergic neurons, but also in GABA-negative neurons, some of which have an unknown neurochemical profile [85]. It thus remains possible that nematodes use glycinergic neurotransmission and that one of the orphan ligand-gated channels in their genome is a glycine receptor [86]. However, if *C. elegans* indeed lacks glycinergic neurotransmission, it was likely lost secondarily, as the wide deployment of glycine as a neurotransmitter in both protostomes and deuterostomes suggests that glycinergic neurotransmission was already present in the last common ancestor of all bilaterian animals. This notion is supported by biochemical evidence for the presence of a functional glycine receptor in the freshwater polyp *H. vulgaris* [29], a cnidarian, and thus a member of one of the earliest-branching metazoan lineages (Figure 1).

### 4.2. Evolution of Glycine Transporters in Deuterostomes: Competing Scenarios

Two glycine transporter genes have previously been described in amphioxus [31], corresponding to our *GlyT* and *GlyT2.1*. We identified an additional gene, which we named *GlyT2.2*, and reconstructed the evolutionary history of glycine transporters using three different tree-building methods (BI, ML, and NJ). Our phylogenies strongly support the split between the GlyT1 and GlyT2 clades. Whereas the GlyT1 clade only contains vertebrate sequences, the GlyT2 clade is composed of sequences from vertebrates and invertebrate deuterostomes. Other invertebrate deuterostome GlyT sequences branch outside the GlyT1 plus GlyT2 clade, indicating that no true GlyT1 orthologs exist in invertebrates, as has previously been suggested [31].

**Figure 8:**
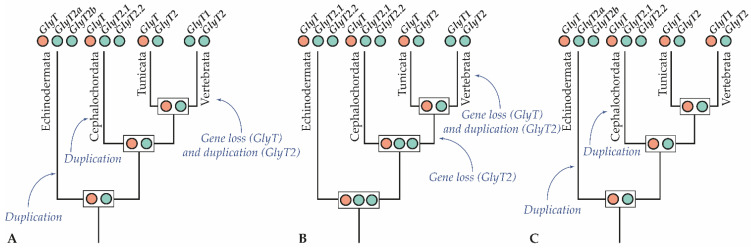
(**A**–**C**) Competing scenarios for the evolution of glycine transporter genes in deuterostomes. Each circle represents a glycine transporter (*GlyT*) gene. Genes of the same color are orthologs. Boxed circles at internal nodes represent ancestral hypothetical glycine transporter complements.

Within the GlyT2 clade, the tunicate plus vertebrate branch is very strongly supported, leaving little doubt that the ascidian tunicate *C. intestinalis* possesses a true GlyT2 ortholog. Conversely, the evolutionary relationships of the other invertebrate GlyT2-like sequences are more difficult to resolve. One possible scenario (Figure 8A) is that an ancestral glycine transporter gene was duplicated early in the deuterostome lineage, before the split of echinoderms and chordates, giving rise to one *GlyT* and one *GlyT2-like* sequence. The *GlyT2-like* gene was then duplicated in the amphioxus lineage, resulting in the *GlyT2.1* and *GlyT2.2* genes, which are located on the same scaffold in the genome, only about 100 kb apart (Table 1). Interestingly, the genome of the sea urchin *S. purpuratus* also contains two *GlyT2-like* genes (at a distance of 16 Mb from each other), which thus also originated by lineage-specific duplication. Another possible scenario for the evolution invertebrate GlyT2-like sequences (Figure 8B) is that ancestral deuterostomes already possessed two *GlyT2* paralogs, one of which was lost in the lineage leading to extant tunicates and vertebrates. However, this scenario is not supported by our phylogenies, as it predicts that sea urchin and amphioxus GlyT2.1 and GlyT2.2 stably associate in the tree. Instead, we found that only the two sea urchin GlyT2 sequences formed a highly supported clade.

Regarding the origin of vertebrate *GlyT1*, one scenario suggested by our phylogenies is that the *GlyT1s* originated by duplication from an ancestral *GlyT2* gene, very early during vertebrate diversification, but after the split from the tunicate lineage [87]. In this scenario, the last common ancestor of tunicates and vertebrates had a *GlyT* gene that was subsequently lost in the lineage leading to extant vertebrates (Figure 8A). An alternative scenario is that the vertebrate *GlyT1s* might be orthologous to the *GlyTs* of invertebrate deuterostomes, as previously suggested [31]. However, this scenario (Figure 8C) can only be reconciled with our phylogenies, if the vertebrate *GlyT1s* have experienced a substantial functional diversification with concomitant changes in their coding sequences. Phylogenetic analyses would thus be unable to correctly assess the orthology of GlyT1 and GlyT sequences. Interestingly, in the ascidian tunicate *C. intestinalis*, *GlyT* and *GlyT2* are both located on chromosome 7, at a distance of about 640 kb from each other [31]. However, while an analysis of genetic synteny of vertebrate glycine transporter loci revealed a general conservation of the genes linked to *GlyT1* and *GlyT2*, analyses of genetic synteny between invertebrate and vertebrate glycine transporter loci remained largely inconclusive [31].

### 4.3. Amphioxus Glycine Transporter Paralogs Are Differentially Expressed in Glia and Neurons

Analysis of the expression patterns of the three amphioxus glycine transporter genes during development revealed that they were already expressed by the mid-neurula stage, which is earlier than what has been reported in vertebrates [13,88,89]. This finding indicates that glycine transporters perform functions other than glycine neurotransmission in amphioxus, a notion supported by the fact that all three amphioxus paralogs are expressed in the pharynx and *GlyT2.1* additionally in the somites.

When comparing the expression domains of the amphioxus glycine transporter genes, that of *GlyT2.2* appears to be the most divergent. Excepting the signal in the anterior cerebral vesicle, *GlyT2.2* is mainly expressed outside of the nervous system. Conversely, both *GlyT* and *GlyT2.1* are widely expressed in the central nervous system, albeit in different cell types: *GlyT* is expressed by ventrolateral neurons, whereas *GlyT2.1* transcripts are mostly localized in floor plate and mediolateral glial cells (Figure 7). This situation is inversed in vertebrates, where *GlyT1* is widely expressed by glial cells and glutamatergic neurons [13,90] and *GlyT2* expression is characteristic of glycinergic neurons [20,91]. This observation supports the notion that the vertebrate *GlyT1* genes derived from the duplication of an ancestral *GlyT2* gene very early during vertebrate diversification (Figure 8A,B). In this scenario, an ancestral chordate *GlyT2* gene was already preferentially expressed in glial cells and, after its duplication in the vertebrate lineage, this same specificity was maintained exclusively in the *GlyT1* paralog. To reconcile this observation with the alternative scenario that the vertebrate *GlyT1s* are orthologous to the *GlyTs* of invertebrate deuterostomes (Figure 8C), one would have to assume that the glycine transporters of the ancestral chordate were not differentially expressed in neurons and glia and that this specialization evolved independently in both the cephalochordate and vertebrate lineages, resulting in different paralogs being expressed in homologous cell types.

### 4.4. Role of Glycine and Glycine Transporters in Larval Swimming

In vertebrates, glycinergic neurotransmission in the brainstem and spinal cord has important roles in escape reflexes and in the modulation of rhythmic motor behaviors, such as locomotion and breathing. Consequently, mutation of genes encoding glycine transporter or receptor subunits results in excessive startle reflexes (hyperekplexia) in humans and swimming-related phenotypes in zebrafish [4,92,93]. Rhythmic motor patterns for locomotion are generated by spinal networks called central pattern generators (CPGs). Spinal CPGs are remarkably conserved in vertebrates, regardless of the type of locomotion (swimming, walking or flying), and have further already been described in larvae of the ascidian tunicate *C. intestinalis* [94,95]. CPGs typically consist of segmentally arranged motor neurons that innervate adjacent trunk muscles, glycinergic commissural interneurons, ipsilaterally-projecting inhibitory interneurons, and excitatory glutamatergic interneurons. Excitatory interneurons project to all other neuronal cell types that establish the CPG, and glycinergic inhibitory interneurons cross the midline to inhibit motor neurons and all interneuron types. In this network, contralateral inhibition by glycinergic interneurons allows the generation of alternating and finely timed motor outputs that result in repetitive movements, such as swimming [96,97]. Indeed, glycine receptor antagonists abolish the alternation between sides [27,98].

The role of glycinergic transmission in the amphioxus swimming CPG has not yet been investigated. However, serial ultrastructural data are available for the anterior part of the amphioxus larva, which have allowed the reconstruction of a putative locomotory control center in the dien-mesencephalic and rhombospinal regions [36,99,100,101]. Startle responses result from the action of ventral compartment (VC) motor neurons [102] that innervate deep muscle fibers responsible for fast swimming. Excitatory signals to VC motor neurons come from ectodermal sensory neurons, anterior bipolar neurons, glutamatergic large paired neurons (LPNs), and cholinergic ipsilateral projecting interneurons (IPNs). Inhibitory inputs come from GABAergic commissural neurons (CNs) and a pair of multipolar neurons (MPs) located at the level of somite 2 [36]. MPs have been mapped to the first pair of *VGAT*+/*GAD*− cells in the rhombospinal region and have hence been considered as putative glycinergic neurons [35,36]. They have axons that branch into an ipsilateral ascending fiber and a descending fiber that further splits into an ipsilateral and a contralateral branch [100]. All fibers emanating from the right MP form numerous synapses, targeting IPNs and CNs in addition to VC motor neurons. Inputs to MPs come from LPNs and small, likely sensory, fibers. Amphioxus larvae thus possess two types of interneurons that can potentially inhibit motor neurons on the contralateral side: GABAergic CNs and glycinergic MPs. Glycinergic contralateral inhibition may thus be part of the swimming CPG in amphioxus larvae, similar to what has been described in ascidian tunicate larvae and vertebrates. Future work aimed at characterizing the swimming CPGs of larval amphioxus will be important not only for the insights it provides into CPG origin, but for what it tells us about the structure and function of the far more complex vertebrate CPGs.

The anatomical organization of the MP axons, highly branched with multiple serial synapses, might be the key for explaining the disequilibrium between the abundance of cells expressing glycine transporters and the paucity of putative glycinergic neurons in the amphioxus nerve cord. If our interpretation is correct, most ventral neurons of the amphioxus nerve cord receive glycinergic innervation, and GlyT thus functions as a postsynaptic transporter, comparable to the role of vertebrate GlyT1 in glutamatergic neurons. Most floor plate and astroglia-like cells, both of which are intimately associated with the neuropile [37], express *GlyT2.1*. In this cellular context, GlyT2.1 is hence likely involved in the modulation of glycinergic signaling, which, again, is similar to the functions of vertebrate GlyT1. Several ectodermal sensory neurons project to the nerve cord [103]. Many of these neurons are GABAergic or glutamatergic [35,54], and the expression pattern of *GlyT* indicates that some of them are glycinergic. Afferent peripheral neurons might thus contribute significantly to increasing the number of glycinergic synapses in the nerve cord. Notably, *GlyT2.1* is also expressed in the somites until the T1 stage, when the embryo is already capable of coordinated swimming in response to external stimuli. It might thus be that the muscle fibers of amphioxus are a direct target of glycinergic neurons during the early development of the locomotory system, as has been hypothesized for the ascidian tunicate tadpole based on glycine receptor expression [27].

Amphioxus late embryos and larvae possess non-neuronal cells that, as with vertebrate astroglia, express *EAAT2*, a glutamate transporter responsible for modulating glutamatergic transmission [37]. We show here that virtually all the astroglia-like cells of amphioxus also express *GlyT2.1*, strengthening their proposed homology to vertebrate astroglia. Moreover, the co-expression of *EAAT2* and *GlyT2.1* in the same cell populations suggests that glycine and glutamate might co-act at the same synapse. This is known to be the case in vertebrates, where glycine is a co-agonist of NMDA glutamate receptors and GlyT1 is found at glutamatergic synapses, where it modulates the function of NMDAR regulating glycine levels in the receptor microenvironment [6,90].

## 5. Conclusions

Glycinergic neurotransmission performs well-established functions in vertebrates, but its role in invertebrates has been badly neglected. Despite the limited number of studies, glycinergic neurotransmission in invertebrates has been implicated in diverse functions, including the coordination of locomotion, blood pressure regulation, and circadian rhythmicity. Some of these functions may be conserved in vertebrates, implicating glycine as part of an ancestral neurochemical toolkit, present in early metazoans and employed widely among taxa for purposes of intercellular communication.

Perhaps significantly, at least two glycine transporter paralogs are present in the genome of all the animal species we investigated. Additionally, though the phylogenetic relationships of glycine transporter family members are complex, there is clear evidence for lineage-specific duplications of glycine transporter genes in several animal phyla. This strongly suggests that no less than two transporters, likely with different kinetic and/or stoichiometric properties, are necessary for effective glycinergic transmission. As in vertebrates, invertebrate glycine transporter paralogs likely exhibit a differential, cell type-specific expression, which is what we observed in amphioxus, where *GlyT* and *GlyT2.1* are widely expressed in the CNS, but differentially in neurons and glia. Our data also suggest that different glycine transporter paralogs are expressed in homologous cell types in amphioxus and vertebrates, which means further studies on echinoderms and tunicates will be needed to fully explain the evolution of glycinergic neurotransmission from invertebrates to vertebrates.

## Figures and Tables

**Table 1:** List of cephalochordate glycine transporter sequences identified in this work (from *Branchiostoma belcheri*, *Branchiostoma floridae*, and *Branchiostoma lanceolatum*). Glycine transporter sequences previously identified by Shpak and coworkers [31] are highlighted with an asterisk. n.a.: data not available.

Name	Species	Genomic Position	GenBank Accession Number	Transcriptome/EST Data
GlyT	*B. belcheri*	Unplaced scaffold	XP_019646809.1 (isoform X1) XP_019646811.1 (isoform X2) XP_019646812.1 (isoform X3)	n.a.
	B. floridae*	Chr 7:197986	XP_035682443	GETA01030290.1
	*B. lanceolatum*	scf42:456448	n.a.	JT884165.1
GlyT2.1	*B. belcheri*	Unplaced scaffold	XP_019644968.1 (isoform X1) XP_019644969.1 (isoform X2)	n.a.
	B. floridae*	Chr 12: 3077852	XP_035694152.1 (isoform X1) XP_035694153.1 (isoform X2) XP_035694154.1 (isoform X3)	n.a.
	*B. lanceolatum*	scf11:2848268	n.a.	JT866905.1 JT890693.1
	*B. belcheri*	Unplaced scaffold	XP_019644970.1 (isoform X1) XP_019644972.1 (isoform X2) XP_019644973.1 (isoform X3)	n.a.
GlyT2.2	*B. floridae*	Chr 12: 2983693	XP_035694157.1	FE595048
	*B. lanceolatum*	scf11:1893629	n.a.	JT855933.1 JT862870.1

## Data Availability

Data are contained within the article or supplementary material.

## Supplementary Materials

The following files are available online at https://www.mdpi.com/article/10.3390/cells10123392/s1, Table S1: PCR conditions, primers, and amplicon length of *Branchiostoma lanceolatum* genes cloned in this study; Table S2: Multiple sequence alignment of amphioxus glycine transporter proteins; File S1: Alignment in FASTA format of amino acid sequences used to generate phylogenetic trees.
