# Supplementary material for: Functional Conservation and Genetic Divergence of Chordate Glycinergic Neurotransmission: Insights from Amphioxus Glycine Transporters
Source: Cells. 2021 Dec 2;10(12):3392. doi: 10.3390/cells10123392 (PMC8699752; doi:10.3390/cells10123392)
Supplement: Supplementary file 1 [file cells-10-03392-s001.zip › Supplementary_Tables.pdf]

Table S1. PCR conditions, primers and amplicon length of *Branchiostoma lanceolatum* genes cloned in this study. bp: base pairs.

**Gene**	**Forward primer/Reverse primer**	**Length**	**Annealing temperature**
GlyT	5′-ACAACGCCTGGAACACCGAGAACT-3′<br>5′-GAAGAAGGCCAGGACCATCAGAGA-3′	911 bp	54°C
GlyT2.1	5′-TCCCCTACCTCGCTTACCAAAATG-3′<br>5′-AGGCGGCAGACAGGGAGTAGAAGA-3′	825 bp	58°C
GlyT2.2	5′-CGGCGGAGGTGCGTTTTTGAT-3′<br>5′-GAGGGGGTAGGTGCCAGCGTAAGA-3′	1159 bp	59°C
VGAT	5′- GGTCCAGTGTTTGTACGAGGA-3′<br>5′-GATCCCACTTCAGCTTCATGT-3′	947 bp	54°C

Each primer set is listed as forward primer in the first and reverse primer in the second line. Each PCR reaction was carried out with 35 thermal cycles.

Table S2. Multiple sequence alignment of amphioxus glycine transporter proteins. Bbelcheri: *Branchiostoma belcheri*; Bfloridae: *Branchiostoma floridae*; Blanceolatum: *Branchiostoma lanceolatum*.

```
                                  1                                                                                                               115
            Bfloridae GlyT    (1) -----------------MESNGDI--RDMKMREASIPLSVQDTDG------EEEEEEFRDEMAKNRGQWGAKLDFMLSMLGYCVGLGNVWRFPYLCYRNGGGAFLIPYLLFLTFA
         Blanceolatum GlyT    (1) -----------------MESNGDINIKDMKMREASIPLSVQDEE--------EEEEEFRDEVAANRGQWGAKLDFMLSMLGYCVGLGNVWRFPYLCYRNGGGAFLIPYLLFLTFA
   Bbelcheri GlyTisoformX1    (1) -----------------MESN-----GEMKMKEASIPLSVQDHE-------EEEEEEFRDDLAKNRGQWGAKLDFMLSMLGYCVGLGNVWRFPYLCYRNGGGAFLIPYILFLTFA
   Bbelcheri GlyTisoformX2    (1) -------------------------------------------------------------------------------------------------------------------
   Bbelcheri GlyTisoformX3    (1) -------------------------------------------------------------------------------------------------------------------
 Bfloridae GlyT2.1isoformX1    (1) ---------------MTGDTPMAGNEQPTELKQLDPDGNYKFENG-------SVAVEDDGDENKERGNWSNKLDFILSCVGYAVGLGNVWRFPYLAYQNGGGAFLVPYTIMLACA
 Bfloridae GlyT2.1isoformX2    (1) ---------------MTGDTPMAGNEQPTELKQLDPDGNYKFENG-------SVAVEDDGDENKERGNWSNKLDFILSCVGYAVGLGNVWRFPYLAYQNGGGAFLVPYTIMLACA
       Blanceolatum GlyT2.1    (1) ---------------MTGDTP---NEQPTELKQLDPDGNYKFENG-------SVAVEDEGDENKERGNWSNKLDFILSCVGYAVGLGNVWRFPYLAYQNGGGAFLVPYTIMLACA
 Bbelcheri GlyT2.1isoformX1    (1) ---------------MTGETPMAGNEQPTELKQLDPDGNYKFENG-------SAVVEDDGDENKERGNWSNKLDFILSCVGYAVGLGNVWRFPYLAYQNGGGAFLVPYTIMLACA
 Bbelcheri GlyT2.1isoformX2    (1) ---------------MTGETPMAGNEQPTELKQLDPDGNYKFENG-------SAVVEDDGDENKERGNWSNKLDFILSCVGYAVGLGNVWRFPYLAYQNGGGAFLVPYTIMLACA
 Bfloridae GlyT2.1isoformX3    (1) ---------------MTGDTPMAGNEQPTELKQLDPDGNYKFENG-------SVAVEDDGDENKERGNWSNKLDFILSCVGYAVGLGNVWRFPYLAYQNGGGAFLVPYTIMLACA
          Bfloridae GlyT2.2    (1) ------------MDNPALQAGDAGNYNATNEPYTNRDGKWKDSYDPEPNAVSQMDLVEDGDENKERGNWSNKADYILSCIGYAVGLGNVWRFPYLAFENGGGAFLIPYIVMLVFA
       Blanceolatum GlyT2.2    (1) ------------MDNPGLQDAGAANYNATNEPYTARDGKWKDSYDPETNAVSQMDLVEDGDENKERGNWSNKADYILSCIGYAVGLGNVWRFPYLAFENGGGAFLIPYIIMLVFA
 Bbelcheri GlyT2.2isoformX1    (1) MVTTTTMTTSIPMGNMTTDPEKQPLPNGPPQTYTSRDGKWKDSYDPEPNAVSQMDLVDDGDENKERGNWSNKADYILSCIGYAVGLGNVWRFPYLAFENGGGAFLIPYIVMLVFA
 Bbelcheri GlyT2.2isoformX3    (1) -------------MDFPDENDVSLLTAERLAKYTSRDGKWKDSYDPEPNAVSQMDLVDDGDENKERGNWSNKADYILSCIGYAVGLGNVWRFPYLAFENGGGAFLIPYIVMLVFA
 Bbelcheri GlyT2.2isoformX2    (1) ------------MDNPGLQAGEAGNYNATNEPYTSRDGKWKDSYDPEPNAVSQMDLVDDGDENKERGNWSNKADYILSCIGYAVGLGNVWRFPYLAFENGGGAFLIPYIVMLVFA
                                  116                                                                                                             230
            Bfloridae GlyT   (91) GIPLFMMEMSFGQYGSLGPLTIWRACPIFKGIGYGMVVVSGLVCIYYNVIIAWTLHFLFSSFTSALPWASCDNAWNTENCTLAAN------------------------------
         Blanceolatum GlyT   (91) GIPLFMMEMSFGQYGSLGPLTIWRACPIFKGIGYGMVVVSGLVCIYYNVIIAWTLHFLFSSFTSALPWATCDNAWNTENCTLAAN------------------------------
   Bbelcheri GlyTisoformX1   (87) GIPLFMMEMSFGQYGSLGPLTIWRACPIFKGIGYGMVVVSGLVCIYYNVIIAWTLHFLFSSFTSALPWASCDNPWNTENCTLAAN------------------------------
   Bbelcheri GlyTisoformX2    (1) ----------------------MPMCTTGTGIGYGMVVVSGLVCIYYNVIIAWTLHFLFSSFTSALPWASCDNPWNTENCTLAAN------------------------------
   Bbelcheri GlyTisoformX3    (1) ----------------------MPMCTTG--IGYGMVVVSGLVCIYYNVIIAWTLHFLFSSFTSALPWASCDNPWNTENCTLAAN------------------------------
 Bfloridae GlyT2.1isoformX1   (94) GLPIFFLELAYGQFSSQGPVGVWKAIPLLQGVGVCMVCVSFLVGIYYNVIIAYALFYLFASFTSYLPWSDCNNPWNTPECATKDACQQTVNATFQNITLGGEMYSMFGLYDENGT
 Bfloridae GlyT2.1isoformX2   (94) GLPIFFLELAYGQFSSQGPVGVWKAIPLLQGVGVCMVCVSFLVGIYYNVIIAYALFYLFASFTSYLPWSDCNNPWNTPECATK-ACQQTVNATFQNITLGGEMYSMFGLYDENGT
       Blanceolatum GlyT2.1   (91) GLPIFFLELAFGQFSSQGPVGVWKAIPLLQGVGVCMVCVSFLVGIYYNVIIAYALFYLFASFTSYLPWSDCDNPWNTPGCATK-ACQQTVNATFQNITLGGEMYSMFGMYDENGT
 Bbelcheri GlyT2.1isoformX1   (94) GLPIFFLELAYGQFSSQGPVGVWKAIPLLQGVGVCMVCVSFLVGIYYNVIIAYALFYLFASFTSYLPWTDCNNPWNTPECATKDACQQTVNATFQNVTLGGELYSMFGLYDENGT
 Bbelcheri GlyT2.1isoformX2   (94) GLPIFFLELAYGQFSSQGPVGVWKAIPLLQGVGVCMVCVSFLVGIYYNVIIAYALFYLFASFTSYLPWTDCNNPWNTPECATK-ACQQTVNATFQNVTLGGELYSMFGLYDENGT
 Bfloridae GlyT2.1isoformX3   (94) GLPIFFLELAYGQFSSQGPVGVWKAIPLLQGVGVCMVCVSFLVGIYYNVIIAYALFYLFASFTSYLPWSDCNNPWNTPECATKDACQQTVNATFQNITLGGEMYSMFGLYDENGT
          Bfloridae GlyT2.2  (104) GLPIFLLETSLGQFASQGPIRVWRCLPLFQGIGYTQVIASALVGIYYNCIIAYTLFYLFSSFTSDLPWRTCDNTWNTGDCVDT---------------------------NSLAN
       Blanceolatum GlyT2.2  (104) GLPIFLLETSLGQFASQGPIRVWRCLPLFQGIGYTQVIASALVGIYYNCIIAYTLFYLFSSFTSDLPWRTCENTWNTADCVDT---------------------------NSLAN
 Bbelcheri GlyT2.2isoformX1  (116) GLPIFLLETSLGQFASQGPIRIWRCLPLFQGIGYTQVIASALVGIYYNCIIAYTLFYLFSSFTSDLPWRTCDNTWNTGDCVDT---------------------------NSLKN
 Bbelcheri GlyT2.2isoformX3  (103) GLPIFLLETSLGQFASQGPIRIWRCLPLFQGIGYTQVIASALVGIYYNCIIAYTLFYLFSSFTSDLPWRTCDNTWNTGDCVDT---------------------------NSLKN
 Bbelcheri GlyT2.2isoformX2  (104) GLPIFLLETSLGQFASQGPIRIWRCLPLFQGIGYTQVIASALVGIYYNCIIAYTLFYLFSSFTSDLPWRTCDNTWNTGDCVDT---------------------------NSLKN
                                  231                                                                                                             345
            Bfloridae GlyT  (176) --HSALDENVTRISPTQEYWNNRVLGISAGIEETGTIQWELALCLLGAWVVVFFCLFKGIKSSGKVVYFTATFPYMMLIVLFLRGVTLEGAGKGLVYYLTPDFSRLADSQVWYDA
         Blanceolatum GlyT  (176) --HSALEENVTRISPTQEYWNNRVLGISSGIEETGTVQWELALCLLAAWVVVFFCLFKGIKSSGKVVYFTATFPYMMLIVLFVRGVTLEGAGKGLVYYLTPDFSRLADSQVWYDA
   Bbelcheri GlyTisoformX1  (172) --HSALEENVTRISPTQEYWNNRVLGISAGIEETGTVQWELALCLLGAWVVVFFCLFKGIKSSGKVVYFTATFPYMMLIVLFLRGVTLEGAGKGLVYYLTPDFSRLADSQVWYDA
   Bbelcheri GlyTisoformX2   (64) --HSALEENVTRISPTQEYWNNRVLGISAGIEETGTVQWELALCLLGAWVVVFFCLFKGIKSSGKVVYFTATFPYMMLIVLFLRGVTLEGAGKGLVYYLTPDFSRLADSQVWYDA
   Bbelcheri GlyTisoformX3   (62) --HSALEENVTRISPTQEYWNNRVLGISAGIEETGTVQWELALCLLGAWVVVFFCLFKGIKSSGKVVYFTATFPYMMLIVLFLRGVTLEGAGKGLVYYLTPDFSRLADSQVWYDA
 Bfloridae GlyT2.1isoformX1  (209) WVNGTYCGDKGRTSPSEDYWNHYALEITPGIHEPGGIKWQLALSLVLAWVIVFLSLCKGVKSSGKVVYFTATFPYFVLVILLIVGVLQDGHLDGVLFFITPKWERLKEAKVWKDA
 Bfloridae GlyT2.1isoformX2  (208) WVNGTYCGDKGRTSPSEDYWNHYALEITPGIHEPGGIKWQLALSLVLAWVIVFLSLCKGVKSSGKVVYFTATFPYFVLVILLIVGVLQDGHLDGVLFFITPKWERLKEAKVWKDA
       Blanceolatum GlyT2.1  (205) WVNGTYCGDKMRTSPSEDYWNHYALEITPGIHETGGVKWQLALSLVLAWVIVFFSMCKGVKSSGKVVYFTATFPYFVLVILLIVGVLQDGHLDGVLFFITPKWERLKEAKVWKDA
 Bbelcheri GlyT2.1isoformX1  (209) WVNGTFCGNKERKSPSEDYWNHYALEITPGIHEPGGVKWQLALSLVLAWVIVFFSLCKGVKSSGKVVYFTATFPYFVLVILLIVGVLQDGHLDGVLFFITPKWERLKEAKVWKDA
 Bbelcheri GlyT2.1isoformX2  (208) WVNGTFCGNKERKSPSEDYWNHYALEITPGIHEPGGVKWQLALSLVLAWVIVFFSLCKGVKSSGKVVYFTATFPYFVLVILLIVGVLQDGHLDGVLFFITPKWERLKEAKVWKDA
 Bfloridae Glyt2.1isoformX3  (209) WVNGTYCGDKGRTSPSEDYWNHYALEITPGIHEPGGIKWQLALSLVLAWVIVFLSLCKGVKSSGKVVYFTATFPYFVLVILLIVGVLQDGHLDGVLFFITPKWERLKEAKVWKDA
          Bfloridae GlyT2.2  (192) WT-G--N-LSDRVSPSEEYWDRYMLSRSSGIGEPVTVKWQLALCLLLAWIVVYFSLIKGIKSSGKVVYFTATFPYVVLLILLIRGVTLDGALEGIKFFIVPKWSQIANAKVWKDA
       Blanceolatum Glyt2.2  (192) WT-G--N-LSDRVSPSEEYWDRYMLSRSAGIGEPVTVKWQLALCLLLAWIVVYFSLIKGIKSSGKVVYFTATFPYVVLLILLIRGVTLDGALEGIKFFIVPKWSQILNAKVWKDA
 Bbelcheri GlyT2.2isoformX1  (204) WT-G--N-ISDRVSPSEEYWDRYMLSRSAGIGEPVTVKWQLALCLLLAWIVVYFSLIKGIKSSGKVVYFTATFPYVVLLILLIRGVTLEGALEGIKFFIVPKWSQIANAKVWKDA
 Bbelcheri GlyT2.2isoformX3  (191) WT-G--N-ISDRVSPSEEYWDRYMLSRSAGIGEPVTVKWQLALCLLLAWIVVYFSLIKGIKSSGKVVYFTATFPYVVLLILLIRGVTLEGALEGIKFFIVPKWSQIANAKVWKDA
 Bbelcheri GlyT2.2isoformX2  (192) WT-G--N-ISDRVSPSEEYWDRYMLSRSAGIGEPVTVKWQLALCLLLAWIVVYFSLIKGIKSSGKVVYFTATFPYVVLLILLIRGVTLEGALEGIKFFIVPKWSQIANAKVWKDA
```

```
                                     346                                                                                                             460
            Bfloridae GlyT  (289) ASQIFYSLGIAFGGTQVMASYNKFNNNTHRDSVFIALSNCCTSVFAGVVVFSILGHMAHKLDMDVKDVVANGPGLVFVAYPEALTLLPVAPLWSVLFFFMIFTVGLDTQFVMLET
         Blanceolatum GlyT  (289) ASQIFYSLGIAFGGTQVMASYNKFNNNTHRDSVFIALTNCCTSVFAGVVVFSILGHMAHKLDMDVKDVVANGPGLVFVAYPEALTLLPVAPLWSVLFFFMIFTVGLDTQFVMLET
   Bbelcheri GlyTisoformX1  (285) ASQIFYSLGIAFGGTQVMASYNKFNNNTHRDSVFIALSNCCTSVFAGVVVFSILGHMAHKLDMDVKDVVANGPGLVFVAYPEALTLLPVAPLWSVLFFFMIFTVGLDTQFVMLET
   Bbelcheri GlyTisoformX2  (177) ASQIFYSLGIAFGGTQVMASYNKFNNNTHRDSVFIALSNCCTSVFAGVVVFSILGHMAHKLDMDVKDVVANGPGLVFVAYPEALTLLPVAPLWSVLFFFMIFTVGLDTQFVMLET
   Bbelcheri GlyTisoformX3  (175) ASQIFYSLGIAFGGTQVMASYNKFNNNTHRDSVFIALSNCCTSVFAGVVVFSILGHMAHKLDMDVKDVVANGPGLVFVAYPEALTLLPVAPLWSVLFFFMIFTVGLDTQFVMLET
Bfloridae GlyT2.1isoformX1 (324) ATQIFYSLSAAWGGLITMASYNRFQNNCYKDTLIVSLMNCSTSIFAGFVIFSILGFMAKQIGVDVDDVAASGPGLAFIAYPEALTKLPVPPIWAILFFLMLLTLGLDTQFAIIET
Bfloridae GlyT2.1isoformX2 (323) ATQIFYSLSAAWGGLITMASYNRFQNNCYKDTLIVSLMNCSTSIFAGFVIFSILGFMAKQIGVDVDDVAASGPGLAFIAYPEALTKLPVPPIWAILFFLMLLTLGLDTQFAIIET
      Blanceolatum GlyT2.1 (320) ATQIFYSLSAAWGGLITMASYNRFQNNCYKDTLIVSLMNCSTSIFAGFVIFSILGFMAKQIGVDVDDVAASGPGLAFIAYPEALTKLPVPPIWAILFFLMLLTLGLDTQFAIIET
Bbelcheri GlyT2.1isoformX1 (324) ATQIFYSLSAAWGGLITMASYNRFQNNCYKDTLIVSLMNCSTSVFAGFVIFSILGFMAKQIGVDVDDVAASGPGLAFIAYPEALTKLPVPPIWAILFFLMLLTLGLDTQFAIIET
Bbelcheri GlyT2.1isoformX2 (323) ATQIFYSLSAAWGGLITMASYNRFQNNCYKDTLIVSLMNCSTSVFAGFVIFSILGFMAKQIGVDVDDVAASGPGLAFIAYPEALTKLPVPPIWAILFFLMLLTLGLDTQFAIIET
Bfloridae GlyT2.1isoformX3 (324) ATQIFYSLSAAWGGLITMASYNRFQNNCYKDTLIVSLMNCSTSIFAGFVIFSILGFMAKQIGVDVDDVAASGPGLAFIAYPEALTKLPVPPIWAILFFLMLLTLGLDTQFAIIET
         Bfloridae GlyT2.2 (303) AAQIFFSLSAAWGGLLTLASYNKFKNNTIHDALIVALTNCATSVFAGFVIFSILGHMALKLNVTVPEVAKSGFGLAFVAYPEALTLLPVSPLWAIIFFLMLFTLGLDSQFTIVET
      Blanceolatum GlyT2.2 (303) AAQIFFSLSAAWGGLLTLASYNKFKNNTIHDALIVALTNCATSVFAGFVIFSILGHMALKLNVTVPEVAKSGFGLAFVAYPEALTLLPISPLWAIIFFLMLFTLGLDSQFTIVET
Bbelcheri GlyT2.2isoformX1 (315) AAQIFFSLSAAWGGLLTLASYNKFKNNTIHDALIVALTNCATSVFAGFVIFSILGHMALKLNLTVPEVAKSGFGLAFVAYPEALTLLPVSPLWAIIFFLMLFTLGLDSQFTIVET
Bbelcheri GlyT2.2isoformX3 (302) AAQIFFSLSAAWGGLLTLASYNKFKNNTIHDALIVALTNCATSVFAGFVIFSILGHMALKLNLTVPEVAKSGFGLAFVAYPEALTLLPVSPLWAIIFFLMLFTLGLDSQFTIVET
Bbelcheri Glyt2.2isoformX2 (303) AAQIFFSLSAAWGGLLTLASYNKFKNNTIHDALIVALTNCATSVFAGFVIFSILGHMALKLNLTVPEVAKSGFGLAFVAYPEALTLLPVSPLWAIIFFLMLFTLGLDSQFTIVET
                                     461                                                                                                             575
            Bfloridae GlyT  (404) CITGICDEFPHIMG-KYKTWVLLVVSVVMYFLGLTCVTNAGMYWLNLMDWYSAGFSLMVLAFFMCVAISWVYGFQRFCKNVQEMIGYQPNYYFKICWAVISPMVLLFIVVFSMVV
         Blanceolatum GlyT  (404) CITGICDEFPHIMQ-KYKTWVLLAVSVGMYLLGLTCVTNAGMYWLNLMDWYSAGFSLMVLAFFLCIAISWVYGFQRFCKNVQEMIGYQPNYYFKICWAVISPMVLLFIVVFSMVV
   Bbelcheri GlyTisoformX1  (400) CITGICDEFPHIMQ-KYKTWVLLVVSVAMYLLGLTCVTNAGMYWLNLMDWYSAGFSLMVLAFFMCVAISWVYGFQRFCKNVQEMIGYQPNYYFKICWAVISPMVLLFIVVFSMVV
   Bbelcheri GlyTisoformX2  (292) CITGICDEFPHIMQ-KYKTWVLLVVSVAMYLLGLTCVTNAGMYWLNLMDWYSAGFSLMVLAFFMCVAISWVYGFQRFCKNVQEMIGYQPNYYFKICWAVISPMVLLFIVVFSMVV
   Bbelcheri GlyTisoformX3  (290) CITGICDEFPHIMQ-KYKTWVLLVVSVAMYLLGLTCVTNAGMYWLNLMDWYSAGFSLMVLAFFMCVAISWVYGFQRFCKNVQEMIGYQPNYYFKICWAVISPMVLLFIVVFSMVV
Bfloridae GlyT2.1isoformX1 (439) VVTTICDTFKIGHAGIKKTLSTLGFCLVMFLLGLLCVTRSGLYWVNLIDNFAASYSLMVIAITEMLGISWVYGINNFCRDIEMMVGFKPGWYWKATWAVISPGLLIFIFIFSLVE
Bfloridae GlyT2.1isoformX2 (438) VVTTICDTFKIGHAGIKKTLSTLGFCLVMFLLGLLCVTRSGLYWVNLIDNFAASYSLMVIAITEMLGISWVYGINNFCRDIEMMVGFKPGWYWKATWAVISPGLLIFIFIFSLVE
      Blanceolatum GlyT2.1 (435) VVTTVCDQFKIKHTGIKKTLSTLGFCILMFLLGLLCVTRSGLYWVNLIDNFAASYSLMVIAITEMLGISWIYGINNFCRDIKMMVGFEPGWYWKATWAVISPGLLIFIFIFSLVE
Bbelcheri GlyT2.1isoformX1 (439) VTTTVCDQFKIAHTGIKKTLSTLAFCIVMFLLGLLCVTRSGLYWVNLIDNFAASYSLMVIAITEMLGISWIYGINNFCRDIEMMVGFKPGWYWKATWAVISPGLLIFIFIFSLVE
Bbelcheri GlyT2.1isoformX2 (438) VTTTVCDQFKIAHTGIKKTLSTLAFCIVMFLLGLLCVTRSGLYWVNLIDNFAASYSLMVIAITEMLGISWIYGINNFCRDIEMMVGFKPGWYWKATWAVISPGLLIFIFIFSLVE
Bfloridae GlyT2.1isoformX3 (439) VVTTICDTFKIGHAGIKKTLSTLGFCLVMFLLGLLCVTRSGLYWVNLIDNFAASYSLMVIAITEMLGISWVYGINNFCRDIEMMVGFKPGWYWKATWAVISPGLLIFIFIFSLVE
         Bfloridae GlyT2.2 (418) VATAICDGWPTLLR-KKKWLVMLCIAVCCYLLAMPCLTHAGIYHVQLIDSYAGTYPLIIVAIMECIGISYLYGLRRFCKDIAMMAGYQPNYYWQANWAFITPALLTFVLIFSFVF
      Blanceolatum GlyT2.2 (418) VATAICDGWPTLLR-QKKWLVMLSIAVCCYLLAMPCLTHAGIYHVQLIDSYAGTYPLIIVAIMECIGISYLYGLRRFCKDIAMMAGYQPNYYWQANWAFITPALLTFVLIFSFVF
Bbelcheri GlyT2.2isoformX1 (430) VATAICDGWPTLLR-QKKWLVMLTISVCCYLLAMPCLTHAGIYHVVLIDSYAGTYPLIIVAIMECIGISYLYGLRRFCKDIAMMAGYQPNYYWQANWAFITPALLTFVLIFSFVF
Bbelcheri GlyT2.2isoformX3 (417) VATAICDGWPTLLR-QKKWLVMLTISVCCYLLAMPCLTHAGIYHVVLIDSYAGTYPLIIVAIMECIGISYLYGLRRFCKDIAMMAGYQPNYYWQANWAFITPALLTFVLIFSFVF
Bbelcheri GlyT2.2isoformX2 (418) VATAICDGWPTLLR-QKKWLVMLTISVCCYLLAMPCLTHAGIYHVVLIDSYAGTYPLIIVAIMECIGISYLYGLRRFCKDIAMMAGYQPNYYWQANWAFITPALLTFVLIFSFVF
                                     576                                                                                                 678
            Bfloridae GlyT  (518) HVPAYYGP-YQYPNWAICIGWFMAMLSIVMVPLFVVLAILKAKG-SFMERLRYACESSDDWGPALVNASSATSSQ---SDEAEKKIPLEDTEGKGSSTYISTI
         Blanceolatum GlyT  (518) HVPAYYGS-YQYPGWAVCIGWLMALMSIVMVPLFVVLAILKAKG-SFMERLRYACESSDNWGPALMNASSAESSQSDE-DEQEKKVPLRDTEGND-NSYISTI
   Bbelcheri GlyTisoformX1  (514) HVPAYYGD-YQYPGWAICIGWFMALLSIIMVPLFAVLAILKAKG-SFMERLRYACESSDDWGPALVSTSSAESSQS----EPEKKVPLQDTEGTG-NSYISTI
   Bbelcheri GlyTisoformX2  (406) HVPAYYGD-YQYPGWAICIGWFMALLSIIMVPLFAVLAILKAKG-SFMERLRYACESSDDWGPALVSTSSAESSQS----EPEKKVPLQDTEGTG-NSYISTI
   Bbelcheri GlyTisoformX3  (404) HVPAYYGD-YQYPGWAICIGWFMALLSIIMVPLFAVLAILKAKG-SFMERLRYACESSDDWGPALVSTSSAESSQS----EPEKKVPLQDTEGTG-NSYISTI
Bfloridae GlyT2.1isoformX1 (554) YKPPTLNETYIYPGWSQAIAWLMVLSGTIWIPVIAIYRVVTSPGDTFMERLRFACKPAPDWGPYLNQHRALDPERYKPKPDPSKQIVNDPYADIGTKI-----
Bfloridae GlyT2.1isoformX2 (553) YKPPTLNETYIYPGWSQAIAWLMVLSGTIWIPVIAIYRVVTSPGDTFMERLRFACKPAPDWGPYLNQHRALDPERYKPKPDPSKQIVNDPYADIGTKI-----
      Blanceolatum GlyT2.1 (550) YTPPTLNDTYIYPGWSQAVAWLMVLFGTIWIPAIAIYRIAVSPGDTFMERLKFACKPAPDWGPYLNQHRELDSERYKPKPDPSKQIVNDPYADVGTKI-----
Bbelcheri GlyT2.1isoformX1 (554) YKAPTLNDTYIYPGWSQAIAWLMVLFGTIWIPAIAIYRIVTSPGDTFMERLKFACKPAPDWGPYLNQHRALDSERYQAKPDPSKQIVNDPYADIGTKI-----
Bbelcheri GlyT2.1isoformX2 (553) YKAPTLNDTYIYPGWSQAIAWLMVLFGTIWIPAIAIYRIVTSPGDTFMERLKFACKPAPDWGPYLNQHRALDSERYQAKPDPSKQIVNDPYADIGTKI-----
Bfloridae GlyT2.1isoformX3 (554) YKPPTLNETYIYPGWSQAIAWLMVLSGTIWIPVIAIYRVVTSPGDTFMERLRFACKPAPDWGPYLNQYRAH--------------------------------
         Bfloridae GlyT2.2 (532) HEDVSYGD-YEYPLWAVTLGNLILVFCAIWIPGVAIFWLIVTPG-SFMERLRKVTSPTDTWGPHLPQHR---DERYANMANPSQGIGAKYTPNDGTKL-----
      Blanceolatum GlyT2.2 (532) HEDVSYGD-YEYPLWAVTLGNLILVFCAIWIPGVAIFWIIVTPG-SFMERLRKVTTPTDTWGPHLARHR---DERYAHMANPSSGIGAKYTPNDGTKL-----
Bbelcheri GlyT2.2isoformX1 (544) HEDVSYGD-YTYPLWAVTLGNLILVFCAIWIPGVAIFWLIVTPG-SFWERLRKVTSPTDTWGPHLPQHR---DERYANMANPSAGIGAKYTPNDGTKL-----
Bbelcheri GlyT2.2isoformX3 (531) HEDVSYGD-YTYPLWAVTLGNLILVFCAIWIPGVAIFWLIVTPG-SFWERLRKVTSPTDTWGPHLPQHR---DERYANMANPSAGIGAKYTPNDGTKL-----
Bbelcheri GlyT2.2isoformX2 (532) HEDVSYGD-YTYPLWAVTLGNLILVFCAIWIPGVAIFWLIVTPG-SFWERLRKVTSPTDTWGPHLPQHR---DERYANMANPSAGIGAKYTPNDGTKL-----
```

Identical amino acid residues conserved in 100% of the sequences are shown in yellow, similar amino acid residues conserved in less than 100% but more than 50% of the sequences are shown in light blue, and similar amino acid residues conserved in less than 50% of sequences are highlighted in green.
